# Convergence on reduced aggression through shared behavioral traits in multiple populations of Astyanax mexicanus

**DOI:** 10.1186/s12862-022-02069-8

**Published:** 2022-10-14

**Authors:** Roberto Rodriguez-Morales, Paola Gonzalez-Lerma, Anders Yuiska, Ji Heon Han, Yolanda Guerra, Lina Crisostomo, Alex C. Keene, Erik R. Duboue, Johanna E. Kowalko

**Affiliations:** 1 Department of Biological Sciences, Lehigh University, 18015 Bethlehem, PA USA; 2 Department of Integrative Biology and Biomedical Sciences, Florida Atlantic University, 33431 Boca Raton, FL USA; 3 Charles E. Schmidt College of Science, Florida Atlantic University, 33431 Boca Raton, FL USA; 4 Program in Integrative Biology and Neuroscience, Florida Atlantic University, 33458 Jupiter, FL USA; 5 Harriet L. Wilkes Honors College, Florida Atlantic University, 33458 Jupiter, FL USA; 6 Department of Biology, Texas A&M, College Station, TX USA

**Keywords:** Cavefish, Astyanax mexicanus, Aggression

## Abstract

**Background:**

Aggression is observed across the animal kingdom, and benefits animals in a number of ways to increase fitness and promote survival. While aggressive behaviors vary widely across populations and can evolve as an adaptation to a particular environment, the complexity of aggressive behaviors presents a challenge to studying the evolution of aggression. The Mexican tetra, *Astyanax mexicanus* exists as an aggressive river-dwelling surface form and multiple populations of a blind cave form, some of which exhibit reduced aggression, providing the opportunity to investigate how evolution shapes aggressive behaviors.

**Results:**

To define how aggressive behaviors evolve, we performed a high-resolution analysis of multiple social behaviors that occur during aggressive interactions in *A. mexicanus.* We found that many of the aggression-associated behaviors observed in surface-surface aggressive encounters were reduced or lost in Pachón cavefish. Interestingly, one behavior, circling, was observed more often in cavefish, suggesting evolution of a shift in the types of social behaviors exhibited by cavefish. Further, detailed analysis revealed substantive differences in aggression-related sub-behaviors in independently evolved cavefish populations, suggesting independent evolution of reduced aggression between cave populations. We found that many aggressive behaviors are still present when surface fish fight in the dark, suggesting that these reductions in aggression-associated and escape-associated behaviors in cavefish are likely independent of loss of vision in this species. Further, levels of aggression within populations were largely independent of type of opponent (cave vs. surface) or individual stress levels, measured through quantifying stress-like behaviors, suggesting these behaviors are hardwired and not reflective of population-specific changes in other cave-evolved traits.

**Conclusion:**

These results reveal that loss of aggression in cavefish evolved through the loss of multiple aggression-associated behaviors and raise the possibility that independent genetic mechanisms underlie changes in each behavior within populations and across populations. Taken together, these findings reveal the complexity of evolution of social behaviors and establish *A. mexicanus* as a model for investigating the evolutionary and genetic basis of aggressive behavior.

**Supplementary information:**

The online version contains supplementary material available at 10.1186/s12862-022-02069-8.

## Introduction

Aggression, defined as behavior that induces harm or damage from one individual to another individual [1–3], is observed across the animal kingdom. Motivation to perform aggressive behaviors can stem from multiple factors, including resource acquisition, establishment of hierarchies, survival and reproductive success [4–7]. While aggression is widespread, aggressive behaviors vary dramatically between species, and within the same species under different ecological contexts [8–10]. Further, aggression is a complex behavior, with agonist interactions often being composed of multiple behavior components that can serve different purposes, including offensive actions like threatening and physically engaging, and defensive actions like retreating and escaping (for examples, see [11, 12]). A central challenge to understanding the mechanisms underlying aggression is defining how these aggressive behaviors evolve in different ecological contexts.

The Mexican tetra, *Astyanax mexicanus*, is a powerful model for investigating the evolution of behavior [13]. *A. mexicanus* is a single species of fish consisting of river-dwelling, eyed surface fish and at least 30 populations of blind cavefish [14, 15]. These cavefish populations have evolved a number of behavioral differences relative to surface fish, including reduced sleep and schooling [16–18], increased vibration-attraction behavior (VAB) for prey detection [19, 20], and reduced aggression [21–24]. *A. mexicanus* are an excellent model for studying the evolution of behavior for many reasons. First, many *A. mexicanus* cavefish populations have evolved independently of each other, providing the opportunity to examine whether cave-associated behaviors have evolved repeatedly [25, 26]. In addition, there now exists a wide array of tools for genetic and neuronal analysis available in *A. mexicanus* [27–31], providing the opportunity to investigate the mechanistic basis of evolved changes in behavior in this species. Here, we define differences in aggression in cavefish from multiple populations relative to surface fish across multiple, ecologically relevant contexts.

Teleost fish are excellent models for studying aggression, as multiple species are aggressive [11, 32–34]. Fish exhibit a number of behaviors during aggressive encounters, including biting, striking, circling, following, escaping, freezing and avoidance [11, 23, 35]. However, whether shared genetic or neural underpinnings underlie the evolution of each of these behaviors, or whether they evolved independently, is unknown. Thus, *A. mexicanus*, which has populations of highly aggressive surface fish that exhibit multiple aggressive behaviors [21, 24], and populations of cavefish which have evolved reductions in aggression [21–24] provides a powerful opportunity to examine whether reductions in aggression evolve through reducing one or all behaviors that compose aggressive encounters, and if the repeated evolution of loss of aggression occurs through the loss of the same behaviors across populations.

Here, we performed detailed behavioral analysis across different contexts to identify and quantify behaviors that occur during social encounters designed to induce aggression in *A. mexicanus* surface fish and cavefish. Specifically, we asked: (1) Does the evolution of aggression in cavefish occur through modulation of all or a subset of the behaviors composing aggressive interactions? (2) Are the behaviors that occur during aggressive encounters repeatedly reduced or lost in multiple, independently evolved cavefish populations? Together, this work contributes to our understanding of how the complex set of behaviors that compose aggression evolve in populations subject to vastly different ecological conditions.

## Materials and methods

### Fish husbandry

All animal husbandry was performed according to methods previously described  [16, 27]. All protocols were approved by the IACUC of Florida Atlantic University. Fish were raised at 23 ± 1 °C. Adult *A. mexicanus* were housed in groups on a circulating filtration system in 18–37 L tanks on a 14:10 h light cycle that was constant through the animal’s lifetime. All fish used in this study were bred and raised in the laboratory. There were no statistical differences between surface fish from Río Choy and Texas lineages, and both populations were used in this study. Cavefish originated from the Pachón, Molino, Tinaja or Los Sabinos caves. All fish were 6 months – 1-year adults, which ranged from 3 to 6 cm in length.

### Resident-intruder assay

All fish assayed for aggression were fed one hour before behavioral acclimation and assayed between Zeitgeber time (ZT) ZT0-ZT6. Aggressive behaviors were quantified using a resident-intruder assay, which was previously shown to induce aggressive behavior in *A. mexicanus* and other vertebrates [21]. Pairs of resident and intruder fish from the same home tank were transferred to individual 2.5 L plastic tanks and acclimated for 18 h in a dedicated behavioral room in which the light: dark cycle was maintained. All pairs of fish were sex- and size-matched. Following acclimation, the intruder fish was transferred to the tank of the resident fish and their interaction was recorded for 1 h using a Microsoft Studio Webcam (#Q2F-00013). All recordings were performed from the front, lateral side of the tank. For recordings in darkness, both the acclimation and assay were performed in the dark. Infrared (IR) lights (850 nM) and cameras that could detect IR light were used during the resident-intruder assay. All resident-intruder recordings were acquired at 15 frames per second using VirtualDub2 (Version 1.10.5), an open-source video-capture and processing utility developed for Microsoft Windows (https://www.virtualdub.org/features.html).

**Table 1:** Definitions for all aggression- and escape-associated behaviors scored in the resident/intruder assay

Behavior	Description
Biting	Focal fish physically makes contact with another fish with its mouth while performing an opening and closing motion with its mouth.
Circling	Both fish engage in a circular motion, typically with one head facing the tail of the other fish and vice versa.
Following	Focal fish follows the trajectory of another fish, which might or might not be escaping.
Escaping	Focal fish accelerates away from the other fish. This could be in response to either following, biting or striking.
Freezing	Focal fish stops moving for greater than 5 s in any position within the tank.
Avoidance	Focal fish localizes in a corner of the tank for greater than 5 s.
Striking	Focal fish accelerates towards another fish ending in contact (but not necessarily biting).

### Novel tank assay

The novel tank assay, a well-established assay for assessing stress-like behaviors in fish [36], was performed on a subset of fish that were subsequently assayed for aggression in light versus dark conditions. All adult fish were of similar size (3–6 cm). Novel tank assays were performed between Zeitgeber (ZT) ZT6-ZT7 (ZT0 = start of the light phase) as previously described[36, 37] with minor modifications. Groups of fish were transferred from their home tanks on the fish system into tanks in a dedicated behavioral room and allowed to acclimate to the room for at least 1 h. Next, each fish was transferred to a 500 mL plastic holding tank for 10-minute acclimation, followed by gentle transfer into a 2.5 L tank containing 2 L of conditioned fish system water. Once transferred, fish were filmed in the light for 10-minutes using a Microsoft Studio Webcam (#Q2F-00013). All novel tank assay recordings were acquired at 30 frames per second using VirtualDub2. After recording behavior, fish were housed individually in their respective tanks for acclimation in the resident-intruder assay.

### Manual Behavior Annotations

We annotated all staged-fights using the Behavioral Observation Research Interactive Software (BORIS) event-logging program [38]. For all annotations, we scored behaviors that occurred during social interactions, focusing on behaviors observed in *A. mexicanus* and other fish species, and our own observations [24, 39, 40] (Table 1). Some behaviors were scored as single events in time (point events = biting, striking, circling) or continuous behavioral events (state events = following, escaping, freezing, avoidance). Individual fish behavior was scored throughout the video to distinguish between resident and intruder fish.

### Data analysis

#### Manual annotation in BORIS for aggression

All data was exported from BORIS as activity plots and time budgets for quantification as text files (*txt). For each behavior, the number of times the behavior happened was recorded, while the total duration (in seconds) was recorded only for the behaviors that had a time component (following, escaping, freezing and avoidance).

#### Automated Tracking for Novel Tank Behavior

The center position of each fish was tracked using automated tracking with Ethovision software, and x-y displacement was calculated across all frames from the 10-minute recordings following previously published protocols using Ethovision XT13 (version 13.0, Noldus, Inc., Leesburg, VA) [37, 41]. To quantify bottom-dwelling for each fish, the arena was divided into three equal sections in Ethovision and the total duration of time spent in the bottom third of the arena was calculated. Ethovision accurately tracked the position of the fish using background subtraction.

Quantifications of all behaviors can be found in the supplementary materials.

### Statistical analysis

We imported all data extracted from BORIS to Prism 9 (GraphPad). All data was tested for normality using Shapiro-Wilk test and parametric (t-tests for 2 group comparisons and One-Way-ANOVA for multiple group comparisons of a single variable) or non-parametric (Mann-Whitney for 2 group comparisons and Kruskal Wallis for multiple group comparisons) tests were used when appropriate, followed by posthoc tests where relevant (Tukey’s test or Dunn’s test). When analyzing more than one variable, such as the case when comparing the variation between light and dark conditions in surface fish versus cavefish populations, we used 2-Way-ANOVAs or Kruskal Wallis. Data was considered statistically significant if p < 0.05 (*), p < 0.01 (**), p < 0.001 (***), p < 0.0001 (****).

We used the Spearman’s rank-order correlation test to measure the association between all aggressive behaviors annotated with bottom-dwelling, and we calculated the rho (r_s_) for each correlation.

Outputs from statistical tests can be found in the supplementary materials.

## Results

### Aggression-associated behaviors observed in surface fish are reduced in Pachón cavefish

**Fig. 1:**
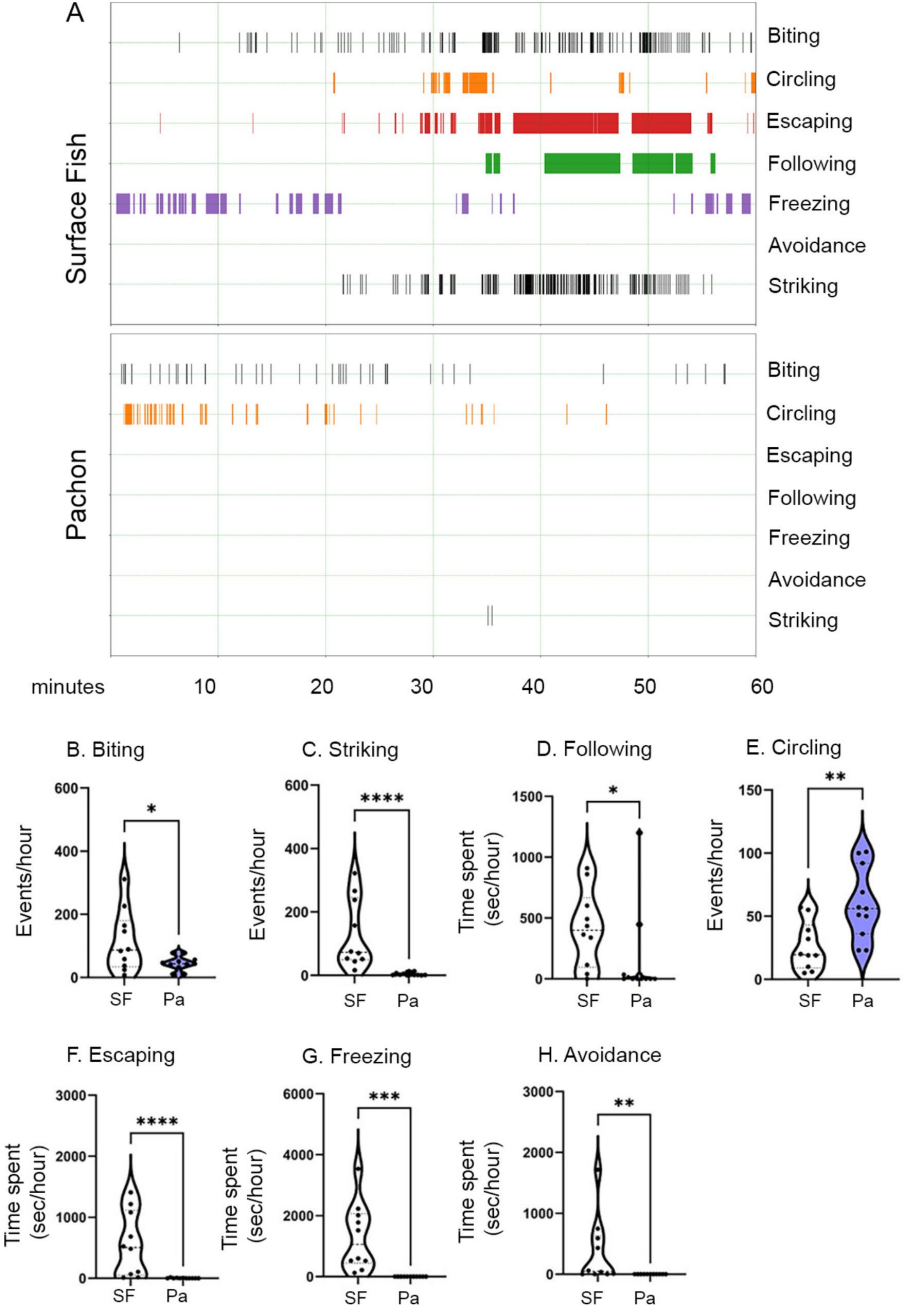
Quantification of social behaviors in the resident/intruder assay for surface fish and Pachón cavefish. (A) Representative ethograms for pairs of surface fish (top) and Pachón cavefish (bottom) during the resident/intruder 1-hour assay. Seven behaviors were annotated: biting, striking, following, circling, escaping, freezing, and avoidance (Table 1) over the 60 min assay period. Behaviors were quantified for each fish, and were pooled for both fish in each resident/intruder assay here (surface: n = 10, Pachón: n = 11). (B-H) Quantifications of behaviors annotated during the resident/intruder assay. All behaviors were scored for both individuals in the tank, and each data point represents either the number of behavioral events (biting (B), striking (C), circling (E)) or the time spent in a behavioral state (following (D), escaping (F), freezing (G), avoidance (H)) for one trial. Unpaired t-tests were calculated for biting (p < 0.05), circling (p < 0.01) and freezing (p < 0.001). Mann-Whitney statistical tests were performed for striking (p < 0.0001), following (p < 0.05), escaping (p < 0.0001) and avoidance (p < 0.01). Significance: p < 0.05 (*), p < 0.01 (**), p < 0.001 (***), p < 0.0001 (****), not significant (ns)

To examine differences in social behavioral evolution between surface fish and cavefish, we first assessed aggressive interactions in surface fish to determine which behavior(s) are displayed during aggressive interactions in surface fish-surface fish resident/intruder assays. Aggression in surface fish was characterized by number of behaviors, including biting, striking, circling, and following (Fig. 1 A). In addition to these aggressive behaviors, surface fish exhibited a number of behaviors typically associated with subordinate/defeated status [11, 42], including escaping, freezing and avoidance (Fig. 1 A). Thus, aggressive interactions in surface fish are composed of multiple aggression-associated and escape-associated behaviors.

To establish whether cavefish evolved reduced aggression through reductions in one or more of these aggressive behaviors, we compared the quantity of each of these aggression- and escape-associated behaviors in surface fish and Pachón cavefish. Pachón cavefish perform fewer aggression-associated behaviors compared to surface fish, including biting (p < 0.05), striking (p < 0.0001), and following (p < 0.05) (Fig. 1B-D). While surface fish also exhibit escape-associated behaviors, escaping (p < 0.0001), freezing (p < 0.001) and avoidance (p = 0.01) were either reduced or absent in Pachón cavefish (Fig. 1 F-H). Interestingly, Pachón cavefish performed significantly more circling than surface fish (p < 0.01) (Fig. 1E), suggesting circling could be an aggression-associated behavior conserved and enhanced in Pachón cavefish, or a social behavior serving another purpose in one or both populations of *A. mexicanus*. We found no statistically significant effect of sex on aggression- or escape-associated behaviors, and no significant interaction between sex and surface fish pairs versus Pachón cavefish pairs for any behavior, except for avoidance. Surface fish males spent more time avoiding compared to surface fish females (Fig S1, Supplementary Data sheets 1&3). Together, this suggests that reduced aggression in Pachón cavefish is characterized by reductions in multiple aggression-associated behaviors observed in surface fish, and a potential shift from aggressive behaviors to an alternative type of social interaction, which includes circling.

As we tracked individual fish during our behavioral annotations, we also examined whether there were quantitative differences in behaviors associated with resident/intruder status. While residents, on average, performed more striking, biting and following than intruders, these differences in behavior did not reach statistical significance. Further, we found no significant effects of resident/intruder status on any aggression- or escape-associated behaviors, or statistically significant interactions between resident/intruder status and population (Fig S2, Supplementary Data sheets 1&3). As we did not find that residents or intruders were more aggressive across trials, we next assessed whether within each of these assays, one of the two fish was more aggressive over the course of the assay, regardless of resident/intruder status. To do so, we designated the fish in each pair that exhibited more strikes the aggressor, and the other fish the non-aggressor. When we compared aggression-associated and escape-associated behaviors for the aggressor versus non-aggressor in surface fish, we found there is a significant asymmetry in most aggression- and escape-associated behaviors in surface fish, with the aggressor performing significantly more biting, striking and following than the non-aggressor, and the non-aggressor performing significantly more escaping, freezing and avoidance than the aggressor (Fig S3A, Supplementary Data sheets 1&4). We observed a similar pattern in Pachón cavefish: Aggressors performed significantly more biting, striking and following, while the non-aggressors performed significantly more escaping (Fig. S3B). Together, these data suggest that, within pairs of both surface fish and Pachón cavefish, one fish is quantitatively more aggressive.

**Fig. 2:**
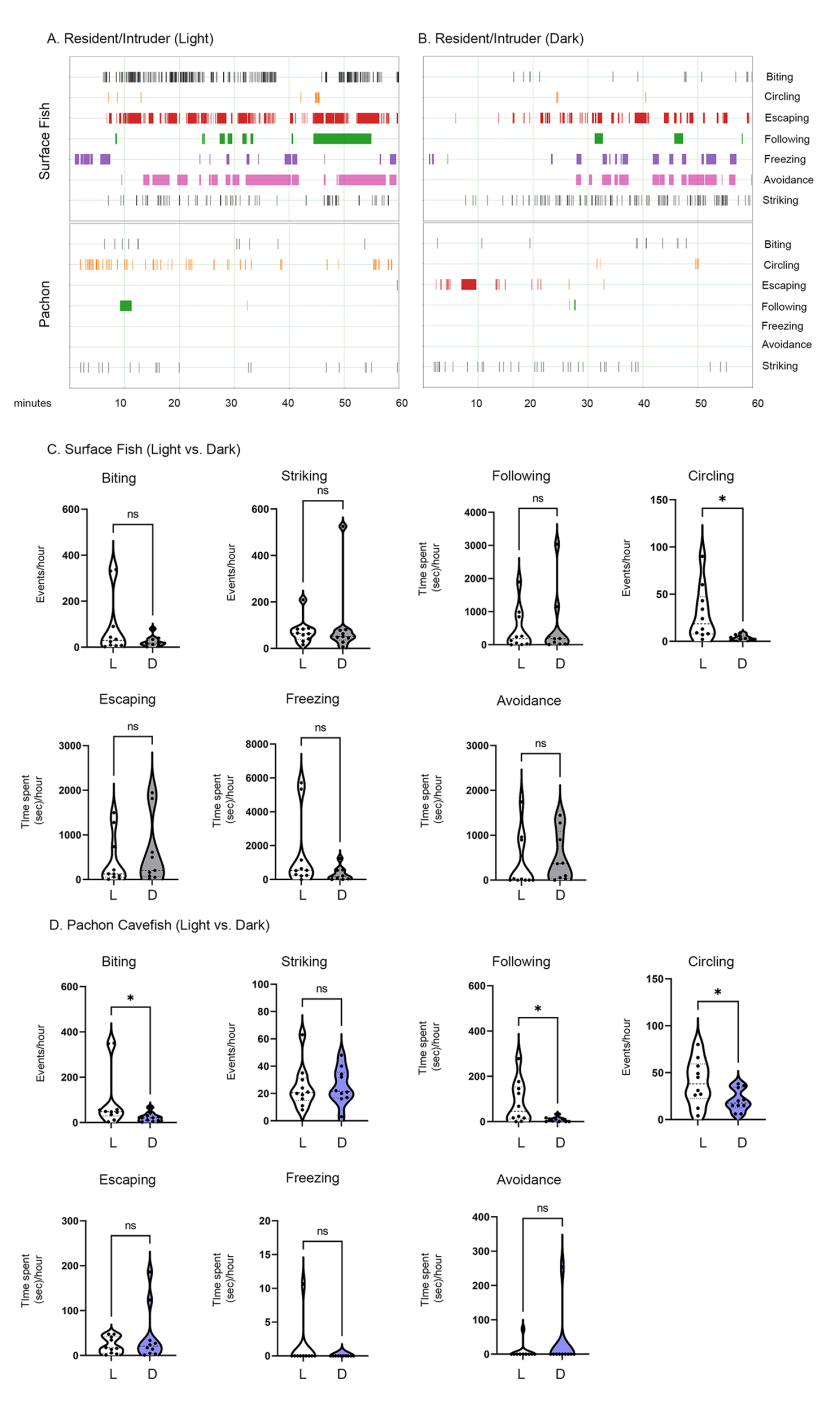
Social Behaviors in a Resident/Intruder Assay Under Light/Dark Conditions. (A-B) Representative merged resident/intruder activity plots for surface fish (top) and Pachón cavefish (bottom) in the light (A) or dark (B) during resident/intruder interactions. (C-D) Quantifications of behaviors annotated during each assay with light (L) versus dark (D) intra-population comparisons for surface fish (C) and Pachón cavefish (D) Assays were performed in the light (surface fish, n = 10; Pachón cavefish, n = 10) and dark (surface fish, n = 9; Pachón cavefish, n = 10). Non-parametric Mann-Whitney tests were performed for all behaviors except for circling (both in C and D), for which an unpaired t-test was performed. Surface fish: biting (p = 0.6461), striking (0.5091), following (p = 0.9682), circling (p < 0.05), escaping (p = 0.6083), freezing (p = 0.1540), avoidance (p = 0.1121); Pachón cavefish: biting (p < 0.05), striking (p = 0.6979), following (p < 0.05), circling (p < 0.05), escaping (p = 0.9765), freezing (p > 0.9999), avoidance (p > 0.9999).p < 0.05 (*), p < 0.01 (**), p < 0.001 (***), p < 0.0001 (****), not significant (ns)

To determine if loss of vision could contribute to the evolution of the reduced aggression- and escape-associated behaviors in cavefish, we performed resident/intruder assays under both light and dark conditions. Surface fish and Pachón cavefish exhibited similar behavior under light and dark conditions for the majority of the behaviors quantified, with no significant differences within populations between striking, escaping, freezing or avoidance (Fig. 2). Biting was reduced in the dark for both populations, but was only significantly reduced for Pachón cavefish (p < 0.05) (Fig. 2CD). Following was only significantly reduced in the dark for Pachón cavefish (p < 0.05) while unchanged for surface fish (Fig. 2CD). Freezing was reduced in the dark in surface fish, but this reduction was not statistically significant (Fig. 2 C). Both surface fish and Pachón cavefish performed less circling in the dark relative to in the light (SF: p < 0.05, Pa:p < 0.05), suggesting that there is an effect of light dependency on this behavior (Fig. 2CD). Thus, surface fish are still able to perform multiple aggression and escape-associated behaviors in the absence of visual cues. This suggests that cavefish did not lose aggression simply due to the loss of the ability to receive visual cues to induce this behavior.

### Differences in aggression between populations are independent of conspecific

**Fig. 3:**
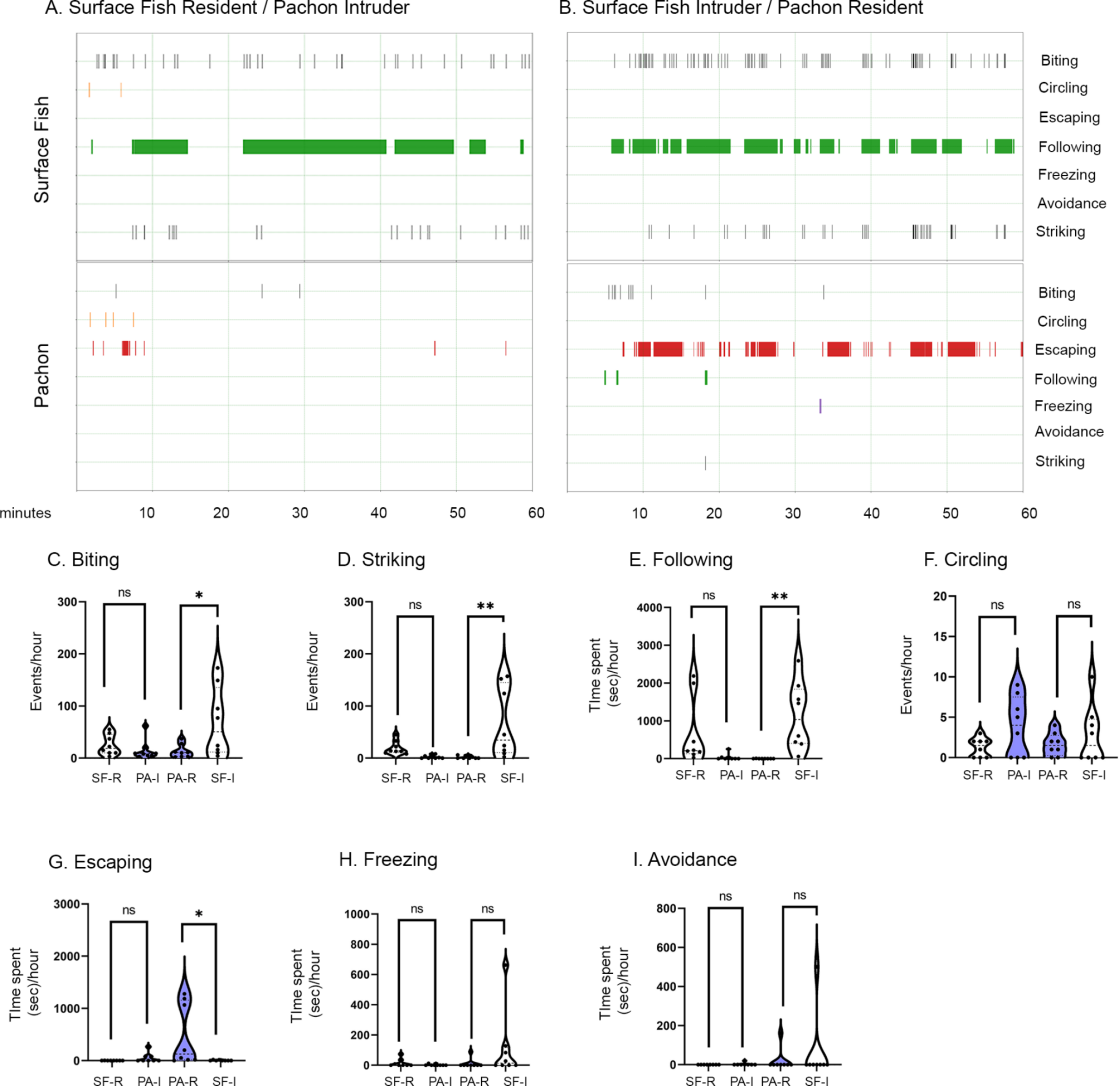
Resident/Intruder dynamics in surface fish versus Pachón cavefish fights. (A-B) Resident/intruder activity plots for surface fish-residents with Pachón-intruders (A) and Pachón-residents with surface fish-intruders (B) during staged fights. (C-I) Quantifications of behaviors annotated during staged fights with resident (R) and intruder (I) intra-population comparisons. 2-Way ANOVAs were performed for all behaviors, followed by Tukey’s multiple comparison’s test for resident versus intruder comparisons: When surface fish were residents: biting (p = 0.9513), striking (p = 0.7403), circling (p = 0.9935), following (p = 0.1689), escaping (p = 0.9865), freezing (p > 0.9999) and avoidance (p = 0.9712). When Pachón cavefish were residents: biting (p < 0.05), striking (p = 0.01), circling (p = 0.8589), following (p = 0.0086), escaping (p < 0.05), freezing (p = 0.2423) and avoidance (p = 0.5680). Significance: p < 0.05 (*), p < 0.01 (**), p < 0.001 (***), p < 0.0001 (****), not significant (ns)

One of the complexities of quantifying aggression is that it involves interactions between multiple individuals. Thus, evolved differences in aggression in cavefish-cavefish interactions could be due to a reduction in aggression in the cavefish aggressor or due to loss of aggression-inducing cues in cavefish. To distinguish between these possibilities, we quantified behavior between inter-population pairs of fish in the resident/intruder assay under two conditions: (1) Surface fish-resident vs. Pachón-intruder, and (2) Pachón-resident vs. Surface fish-intruder. Surface fish exhibited aggression-associated behaviors when paired with a Pachón cavefish opponent (Fig. 3 A-B), suggesting aggression is not associated with the identity of the contender. These interactions induced one escape-associated behavior in Pachón cavefish, escaping (Fig. 3G). When surface fish were residents, they performed more striking and following than Pachón cavefish intruders, but this difference did not reach statistical significance (Fig. 3D, E). By contrast, when Pachón cavefish were the residents, most of the behavioral differences between resident and intruder observed were significant, with surface fish intruders biting (p < 0.01, Fig. 3 C), striking (p < 0.01, Fig. 3D) and following (p < 0.001, Fig. 3E) more, while escaping less (p < 0.01, Fig. 3G) than Pachón cavefish residents. Further, freezing and avoidance were mostly not present throughout these inter-population experiments, and circling was not significantly different between populations (Fig. 3 F, H-I). Taken together, this suggests that surface fish remain aggressive when opposed to cavefish, and that these differences in aggression between fish from different populations are more pronounced when surface fish are the intruders. Although Pachón cavefish do not become aggressive when opposed to a surface fish opponent, their interaction with surface fish induced escape-like responses, reminiscent of the profile of less-aggressive fish during surface fish contests (Fig S3). These results suggest that evolved reductions in cavefish are due to reductions in the behavior of the aggressor, rather than a loss of aggression-inducing cues.

### Stress is unrelated to the quantity of aggressive behaviors in surface fish

**Fig. 4:**
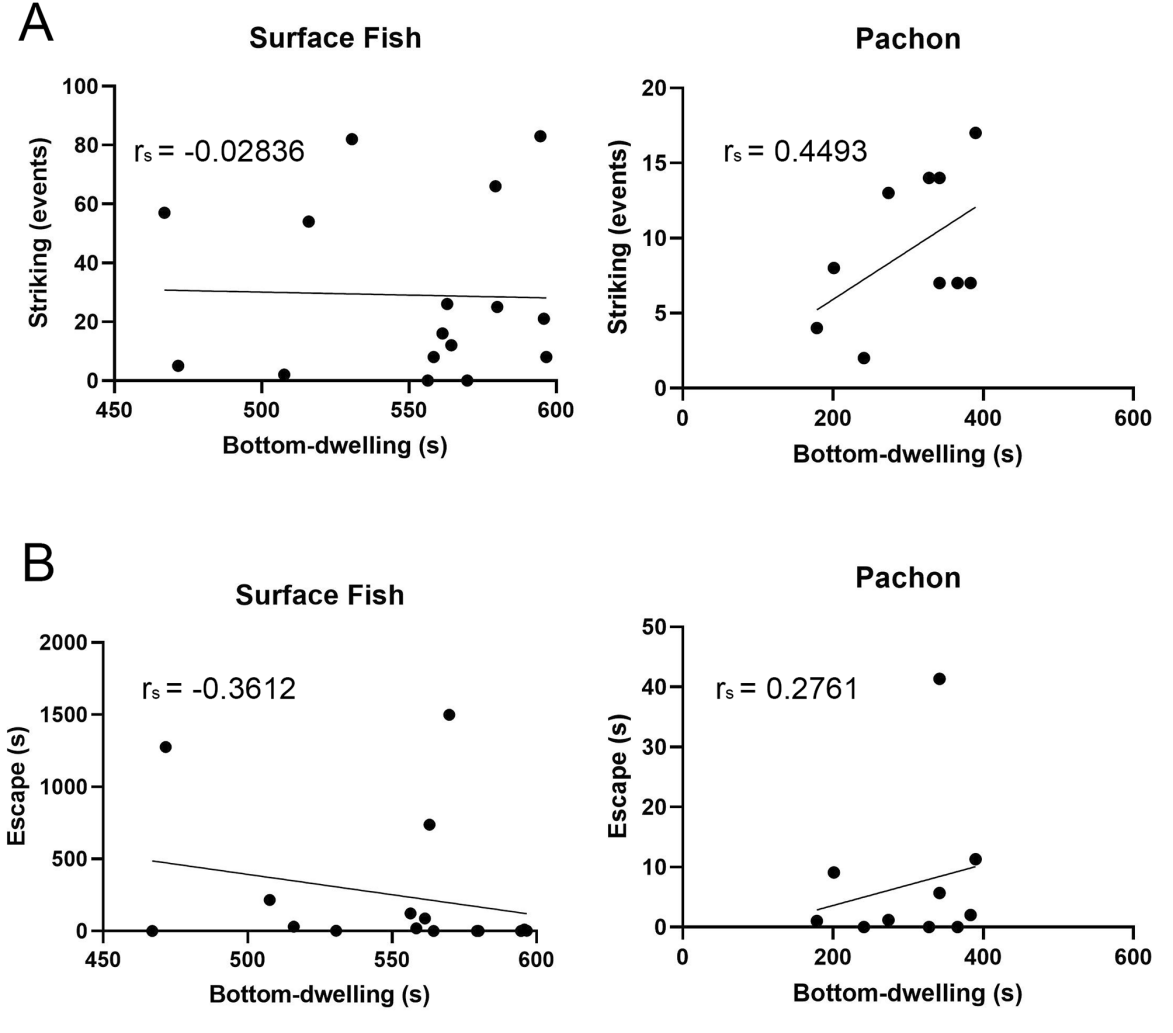
Correlation between two social behaviors during a resident-intruder assay and bottom-dwelling. Correlations between number of strikes and time escaping during the resident/intruder assays and time spent in the bottom third of the tank in the novel tank assay were performed using Spearman’s rank correlation test for striking (A, surface, p = 0.9170, Pachón, p = 0.1941) and escaping (B, surface, p = 0.1694, Pachón, p = 0.4416)

Across multiple species, stress promotes the expression of aggression [43–45]. Both stress-like and aggressive behaviors are reduced in Pachón cavefish [37], raising the possibility of an interaction between these traits. To test whether stress levels influenced levels of aggression, we subjected surface fish and Pachón cavefish to an assay that has been used to quantify stress-like behaviors in multiple fish species [46–48, 36, 49], the novel tank assay prior to the resident/intruder assay acclimation for the comparisons of aggression in the light and the dark (Fig. 2). As a proxy for stress, we measured the amount of time spent bottom-dwelling upon introduction to a novel environment, which was previously reported as a behavior exhibited when fish are stressed [50]. Surface fish spent significantly more time at the bottom of the tank relative to cavefish (Fig S4). These observations confirmed previous findings that suggest surface fish display more stress-like behaviors than cavefish [37]. To examine whether some individuals within each of these populations exhibited more aggression-associated behaviors because they were more stressed, we compared the amount of time spent bottom-dwelling in the novel tank assay with the number of the aggression- or escape-associated behaviors we observed in fish in the light. We found no significant correlations between bottom dwelling and any of the aggression- or escape-associated behaviors in either surface fish or in cavefish (Fig. 4 and Fig.S5). Taken together, aggression appears to be unrelated to stress profile, as quantified by assaying stress-like behaviors, within parental populations of fish, which suggests that differences in stress between individual cavefish and surface fish do not drive the observed differences in aggression within populations.

### Evolution of aggression in cavefish occurs through changes in different aggression-associated behaviors in independently evolved cave populations

**Fig. 5:**
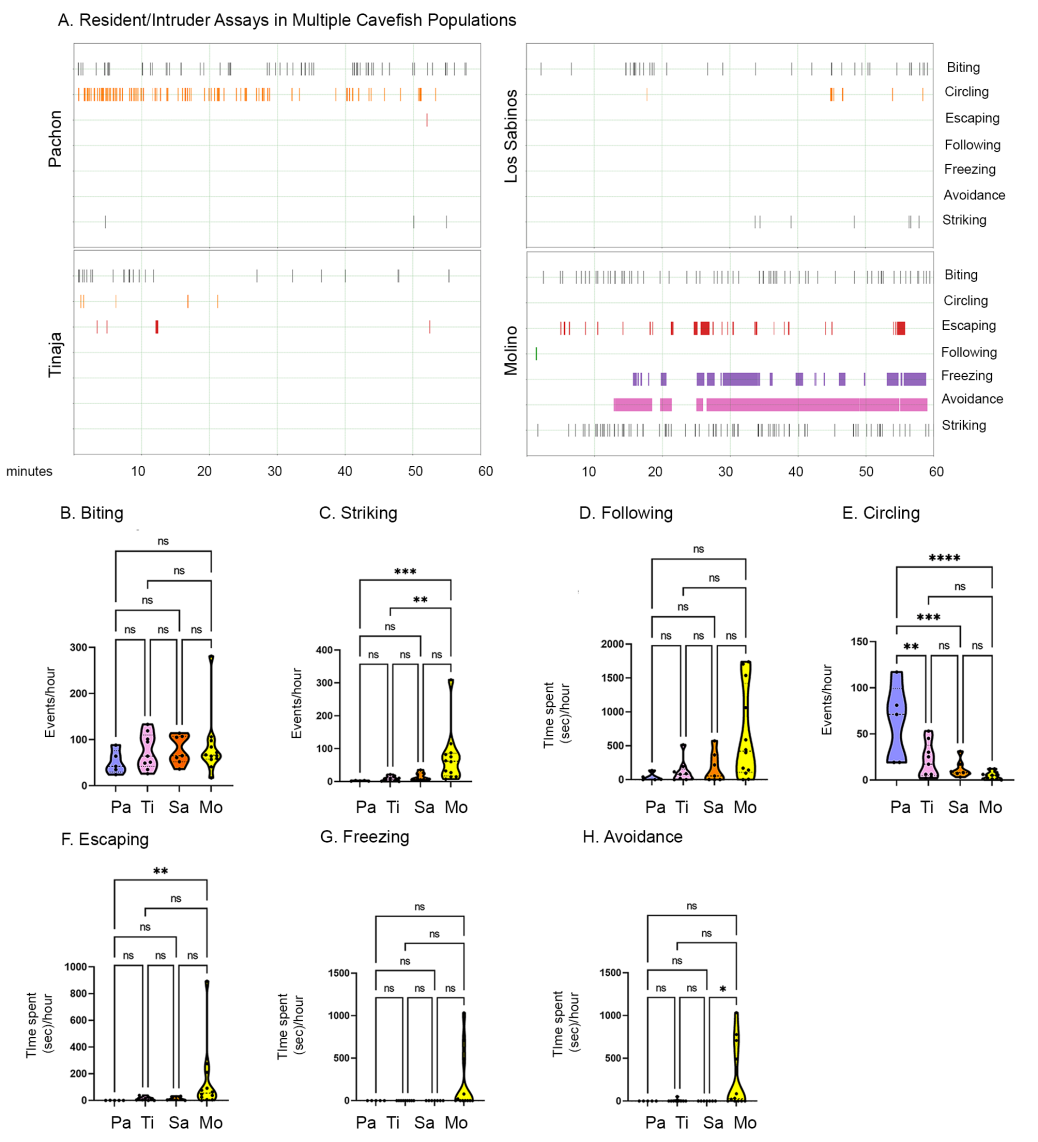
Social behaviors in a resident/intruder assay across multiple cave populations. (A) Representative resident/intruder activity plots for Pachón (top left), Tinaja (bottom left), Los Sabinos (top right) and Molino (bottom right) cavefish during the resident/intruder assay. The number of total behaviors for both the resident and the intruder were combined. All fish were sex and size matched, and sex was not used as a variable given the lack of effect of sex on seven behaviors in Pachón cavefish (Fig S1). (B-H) Quantifications of behaviors annotated during fights with comparisons across populations (Pachón = Pa, Tinaja = Ti, Los Sabinos = Sa, and Molino = Mo). One-way ANOVA followed by Tukey’s multiple comparisons test was performed for circling (Pachón-Molino, p < 0.0001, Pachón-Tinaja, p < 0.05, Pachón-Los Sabinos, p < 0.001, Molino-Tinaja, p = 0.2887, Molino-Los Sabinos, p = 0.885, Tinaja-Los Sabinos, p = 0.7954). Kruskal-Wallis with Dunn’s multiple comparisons test were performed for biting (Pachón-Molino, p = 0.9213, Pachón-Tinaja, p > 0.9999, Pachón-Los Sabinos, p = 0.7564, Molino-Tinaja, p > 0.9999, Molino-Los Sabinos, p > 0.9999, Tinaja-Los Sabinos, p > 0.9999), striking (Pachón-Molino, p < 0.001, Pachón-Tinaja, p > 0.9999, Pachón-Los Sabinos, p = 0.2443, Molino-Tinaja, p = 0.0057, Molino-Los Sabinos, p = 0.3528, Tinaja-Los Sabinos, p > 0.9999), escaping (Pachón-Molino, p < 0.01, Pachón-Tinaja, p = 0.409, Pachón-Los Sabinos, p = 0.4466, Molino-Tinaja, p = 0.206, Molino-Los Sabinos, p = 0.341, Tinaja-Los Sabinos, p > 0.9999), following (Pachón-Molino, p = 0.0585, Pachón-Tinaja, p > 0.9999, Pachón-Los Sabinos, p > 0.9999, Molino-Tinaja, p = 0.2307, Molino-Los Sabinos, p = 0.3641, Tinaja-Los Sabinos, p > 0.9999), freezing (Pachón-Molino, p = 0.1938, Pachón-Tinaja, p > 0.9999, Pachón-Los Sabinos, p > 0.9999, Molino-Tinaja, p = 0.0586, Molino-Los Sabinos, p = 0.0995, Tinaja-Los Sabinos, p > 0.9999), and avoidance (Pachón-Molino, p = 0.0706, Pachón-Tinaja, p > 0.9999, Pachón-Los Sabinos, p > 0.9999, Molino-Tinaja, p = 0.0702, Molino-Los Sabinos, p = 0.0289, Tinaja-Los Sabinos, p > 0.9999). Significance: p < 0.05 (*), p < 0.01 (**), p < 0.001 (***), p < 0.0001 (****), not significant (ns)

*A. mexicanus* cavefish provide a powerful opportunity for studying repeated evolution, as multiple cavefish populations exist that have independently evolved a number of traits [13, 26, 51]. As we found that Pachón cavefish have evolved reduced aggression through reductions in multiple aggression-associated behaviors, we next asked whether other cave populations with differences in ecology and evolutionary history have reduced aggression through reductions in the same or different aggression-associated behaviors. We found that Tinaja and Los Sabinos cavefish exhibited patterns of aggression- and escape-associated behaviors similar to those found in Pachón cavefish, with biting, striking, following, escaping, freezing and avoidance occurring at similar levels between all three of these cavefish populations (Fig. 5). However, Molino cavefish exhibited a different set of behaviors compared to fish from these three cavefish populations. Specifically, Molino cavefish displayed statistically significantly more striking and escaping compared to Pachón cavefish, and trended towards more following, freezing and avoidance relative to other cave populations (Fig. 5). Further, the increase in circling behavior we observed in Pachón cavefish relative to surface fish (Fig. 1E) was not present in other cavefish populations (Fig. 5 A, E). Taken together, these results suggest that different cavefish populations have evolved differences in aggression through alterations to different subsets of aggression- and escape-associated behaviors.

## Discussion

Aggression is a complex behavior that serves multiple purposes throughout the animal kingdom. It is often composed of multiple behavioral components, and different species can exhibit both different levels of aggression, as well as different subsets of the behavioral components that together compose aggressive interactions [11, 12, 52, 53]. Thus, understanding how different patterns of aggressive behaviors evolve in different ecological contexts is critical for understanding the genetic and neural mechanisms contributing to evolution of these complex social behaviors. Here, through systematic quantification of behaviors during aggressive interactions, we find that many of the behaviors observed in other teleost fish are also observed during aggressive encounters in surface *A. mexicanus*. This is in line with previous qualitative characterizations of aggression in *A. mexicanus*, which report that multiple aggressive behaviors are observed during aggressive interactions in surface fish, including ramming (equivalent to striking here), circling and biting [24]. However, some behaviors previously observed in surface *A. mexicanus*, including fin-spreading and snake-swimming [24], were not observed here. This may be due to differences in assay conditions. Previous work used larger tanks (7 L) with groups of up to four fish, and observed behavior across multiple days [24]. Here, in addition to identifying different aggressive behaviors, we quantified each of these aggressive behaviors in surface fish and cavefish from multiple cave populations. This analysis revealed that reduced aggression has evolved in at least three cavefish populations of *A. mexicanus* through reductions in multiple, though not all, aggression- and escape-associated behaviors, and identified a population of cavefish that has elevated levels of a subset of aggression- and escape-associated behaviors relative to other cavefish populations. Together, these results suggest that different genetic mechanisms may underlie the evolution of different aggression-associated behaviors within cave populations. Further, they suggest that reduced aggression has evolved multiple times, and through modulation of some similar and some different social behaviors in different cavefish populations.

External factors from the environment can play a role in levels of aggression exhibited by individuals [54, 55]. Here, we examined whether morphological alterations in cavefish, specifically loss of eyes and vision contribute to the evolution of these differences in aggressive behaviors. In other fish species, aggression is reduced when light intensity is decreased, or when fish are placed in the dark [56, 57], demonstrating the importance of visual cues for inducing aggression. In *A. mexicanus*, there is some degree of controversy regarding the presence of aggression in the dark, as some studies report reduced aggression in surface fish in the dark [23], whereas others found that vision was dispensable for aggression in sighted surface fish [21], and that surface fish raised following a lensectomy early in development are highly aggressive [22]. Our findings were in line with this latter work. Further, while previous work quantified aggression under light and dark conditions as a single metric (striking/attacking), here, we found that multiple aggression-associated behaviors are observed under dark conditions in surface fish. Together, this suggests that observed reductions in aggression in cavefish are unlikely to be due simply to the evolution of eye regression.

The differences in aggression observed here could be mediated through the evolution of other sensory systems or sensory processing in cavefish. Cavefish and surface fish both produce and respond to sounds made by other fish. However, there is evidence that sounds produced by these fish can be produced under different social contexts, and can elicit different responses in Pachón cavefish compared to surface fish. For example, a sound produced by surface fish during aggressive encounters is similar to a sound produced by Pachón cavefish under conditions that induce foraging behavior, suggesting that the same sounds may be used for different purposes in this species [58]. Whether these differences in acoustic communication exist in other cavefish populations is currently unknown. Further, other sensory systems differ between cavefish and surface fish, and could modulate aggression. For example, the lateral line is enhanced in some cavefish populations, including Pachón and Tinaja, with fish from both of these populations having more lateral line mechanosensory neuromasts relative to surface fish [59, 60]. The lateral line plays a role in social interactions in the absence of visual cues in surface fish [60], however, whether these evolved differences in the lateral line contribute to differences in aggression is currently unknown.

Circling behavior has been associated with aggression in other fish species, including zebrafish[11, 61] and sound-producing piranhas [62]. Our results were intriguing in that Pachón cavefish perform fewer of all aggression- and escape-associated behaviors except for circling. When compared with other cave populations, including Molino cavefish which exhibit aggression-associated behaviors, we found that increased circling is unique to Pachón cavefish. This behavior may not necessarily be aggression-associated or social behavior-associated, but instead serve a different purpose in this species. For example, previous reports have found that Pachón cavefish perform stereotypic repetitive circling, and that this behavior decreases under conditions that increase social interactions [63]. Further, circling could serve as an exploratory behavior in cavefish, which could result in context specific differences in circling behavior, for example, between resident versus intruder fish, which would be difficult to detect under the current assay conditions. Alternatively, it has been suggested that cavefish may have evolved a different type of aggressiveness, such as aggressive-associated behaviors related to foraging rather than dominance or territoriality [21, 35]. If cavefish have indeed evolved to exhibit these aggressive behaviors when foraging, the increase in circling observed here may correspond to this foraging-related aggression. Thus, increased circling in Pachón cavefish may indicate an evolved shift from dominance-associated aggression to an alternative type of social interaction. We hypothesize that the circling behavior that we observed is unrelated to dominance- or territorial-based aggression, due to it being observed more often in Pachón cavefish, which have evolved reductions in the other aggression-associated behaviors. Whether it is an exploratory behavior, foraging associated, or serving a different purpose is outside of the scope of this study, but would be interesting to explore in future studies through examining circling behavior in fish that are in novel or familiar environments, as well as under fed and starved conditions.

Previous work suggests that aggression and stress could modulate each other in other fish species [43, 45]. For example, in zebrafish, unpredictable chronic stress (UCS), as well as increases in stress-associated cortisol levels, increased aggression in male fish [43]. In *A. mexicanus*, stress-like behaviors measured in a novel-tank assay are reduced in the multiple populations of cavefish, including Pachón, Tinaja and Molino cavefish, relative to surface fish [37]. Although chemical signals such as cortisol levels may provide a direct measure of stress, pharmacological studies have validated a novel tank assay as an efficient way to identify behavioral responses to stress [64]. Thus, we used this as a behavioral read-out of the “stress-like” status of a fish prior to a resident/intruder assay. We found that intra-population differences in stress-levels were not correlated with levels of aggression- or escape-associated behaviors in either cavefish or in surface fish. This suggests that, within *A. mexicanus* populations, individual differences in stress do not predict levels of aggression. Whether evolved differences in response to a stressful environment between populations is related to the evolution of reduced aggression in cavefish of this species remains to be determined.

Loss of aggression is observed in other animals that have evolved to live in cave environments, including other cavefish [65] and other cave species, like the whip spider *Phrynus longipes* [66]. However, some cave animals are aggressive (for example [67]). Thus, ecological factors beyond living in the dark may play a role in the evolution of aggressive behaviors in cave populations. To determine if and how aggression-associated behaviors have evolved across closely related cave populations, we examined whether repeated loss of aggression-associated behaviors has evolved in multiple cave populations of *A. mexicanus.* Studies in *A. mexicanus* suggest that cave populations are derived from at least two colonization events [68–71]. Surface fish previously inhabiting the Sierra de El Abra region gave rise to a southern lineage of cavefish, including Pachón, Los Sabinos, Tinaja and others [72], while another lineage of surface fish gave rise to the northern populations of cavefish, including Molino and Escondido [72]. Genetic studies suggest that many traits have evolved repeatedly in these different cave populations, whether they derive from these different colonization events, or even between cave populations from the El Abra caves. These traits include genetically encoded morphological traits such as the size of the eye primordia [73, 74], and behavioral traits, including foraging behaviors [75]. Our work suggests that there is independent loss of aggression in multiple cavefish populations of *A. mexicanus*, with multiple aggression- and escape-associated behaviors reduced in three cave populations. However, not all cavefish populations evolved the same reductions in aggression-associated behaviors (e.g., circling), suggesting reduced aggression has evolved independently in these different populations, and that reductions in overall aggression do not need to occur through reductions in all of the same behaviors. Further, Molino cavefish show higher levels of at least one aggression-associated and one escape-associated behavior relative to Pachón cavefish, in line with a previous study that found that Molino cavefish are aggressive [22]. These results demonstrate that even among *A. mexicanus* cavefish, reductions in all aggression-associated behaviors have not evolved in all cave populations. The maintenance of some aggression-associated behaviors in Molino cavefish could be due to the ecological environment of the Molino cave favoring the conservation of aggression- and escape-associated behaviors. Molino fish display phenotypes intermediate to surface fish and Pachón cavefish for a number of traits [75, 76], supporting differences in evolutionary history of this population, or ecology of this cave relative to the Sierra de El Abra cavefish populations.

Ultimately, these findings pose several new questions: (1) While recent work has demonstrated that other populations from Sierra de Guatemala are aggressive [77], are these conserved aggressive behaviors specific to the cavefish derived from this colonization, or are other, currently untested cavefish populations from the Sierra de El Abra aggressive? (2) Do the same genes underlie reduced aggression in the Pachón, Tinaja and Los Sabinos populations? Sampling fish from more caves will provide answers to some of these questions. Further, identifying and functionally interrogating the genes that are contributing to the loss of aggression in *A. mexicanus* will provide additional insight into the genetic factors contributing to natural variation in aggression in this species. Methods such as QTL analysis and functional interrogation of candidate genes using CRISPR-Cas9 that are available in this species could be used in the future to answer these questions [28, 78–80]. This work provides a platform for investigating the extent to which heredity and/or environmental pressures inform the evolution of aggression across closely related populations in a same species.

## Electronic supplementary material

Below is the link to the electronic supplementary material.

Supplementary Material 1

Supplementary Material 2

Supplementary Material 3

Supplementary Material 4

Supplementary Material 5

Supplementary Material 6

## Data Availability

All data generated or analyzed during this study are included in this published article in the following supplementary files: Supplementary Data Sheet 1, Supplementary Data Sheet 2, and Supplementary Data Sheet 3. All raw videos are available upon request from the authors (contact: Johanna Kowalko, jok421@lehigh.edu).
