# Supplementary material for: Convergence on reduced aggression through shared behavioral traits in multiple populations of Astyanax mexicanus
Source: BMC Ecol Evol. 2022 Oct 14;22:116. doi: 10.1186/s12862-022-02069-8 (PMC9563175; doi:10.1186/s12862-022-02069-8)
Supplement: Supplementary file 2 — Supplementary Material 2 [file 12862_2022_2069_MOESM2_ESM.pdf]

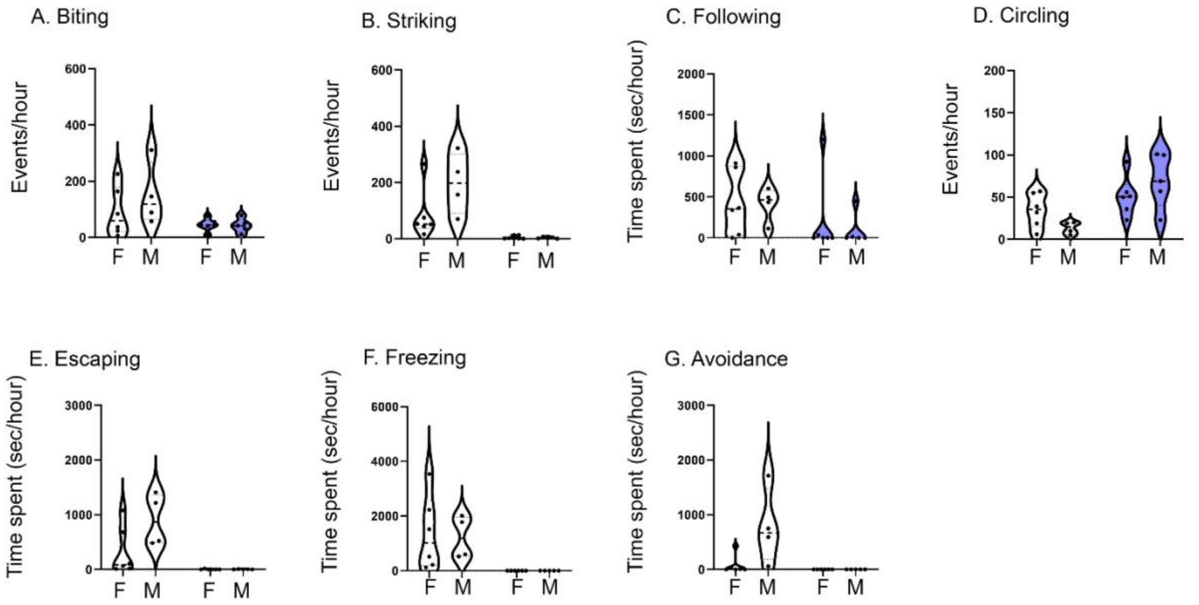

Supplemental Figure 1. Comparison of aggressive- and escape-associated behaviors in male versus female fish. Quantification of behaviors annotated during the resident/intruder assay in the light with female (F) versus male (M) intra-population comparisons. Fish in trials were sex-matched, and trials of males and females presented here were pooled in Figure 1. No significant effects of sex or sex by population interactions were found using a two way ANOVA for any behavior except for avoidance. All statistics can be found in the supplemental data sheet 3.

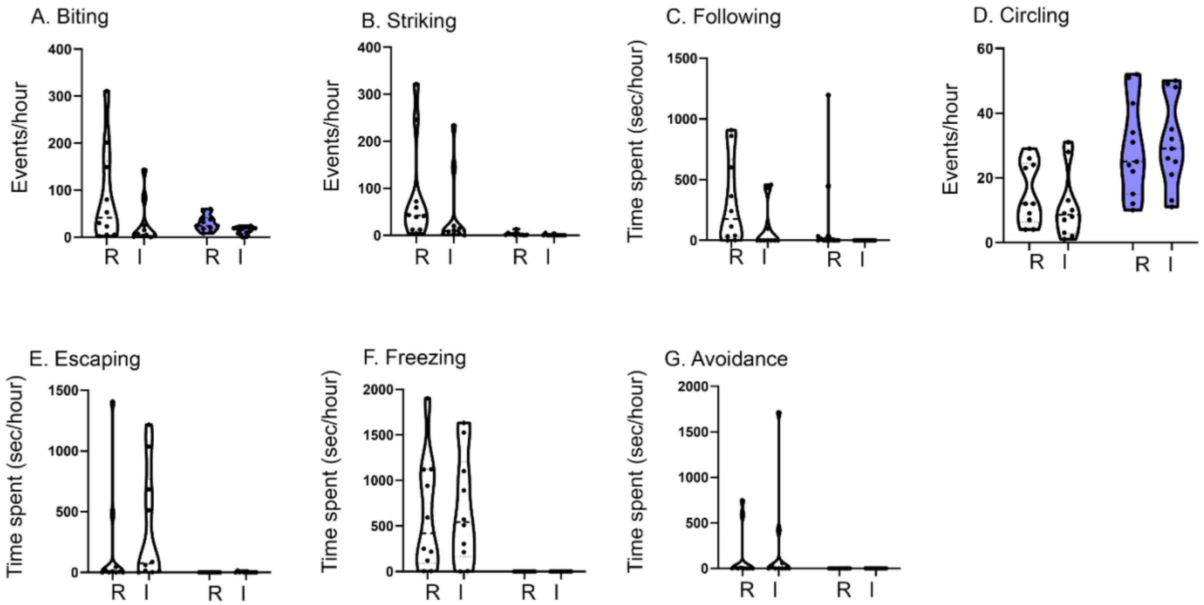

Supplemental Figure 2. Patterns of Aggression in Residents v Intruders. Quantification of behaviors annotated during the resident/intruder assay in the light with resident (R) versus intruder (I) intra-population comparisons. All behaviors were scored for both individuals in the tank, and each data point represents the number of behavioral events (biting (A), striking (B), circling (D)) or the time spent in a behavioral state (following (C), escaping (E), freezing (F), avoidance (G)) for one trial for individual fish. Pooled results can be found in main figure 1. Surface fish (n = 10 residents, 10 intruders) are represented in white, while Pachón cavefish (n = 11 residents, 11 intruders) are represented in light blue. No significant differences between residents and intruders were found using a two way ANOVA. All statistics can be found in supplemental data sheet 3.

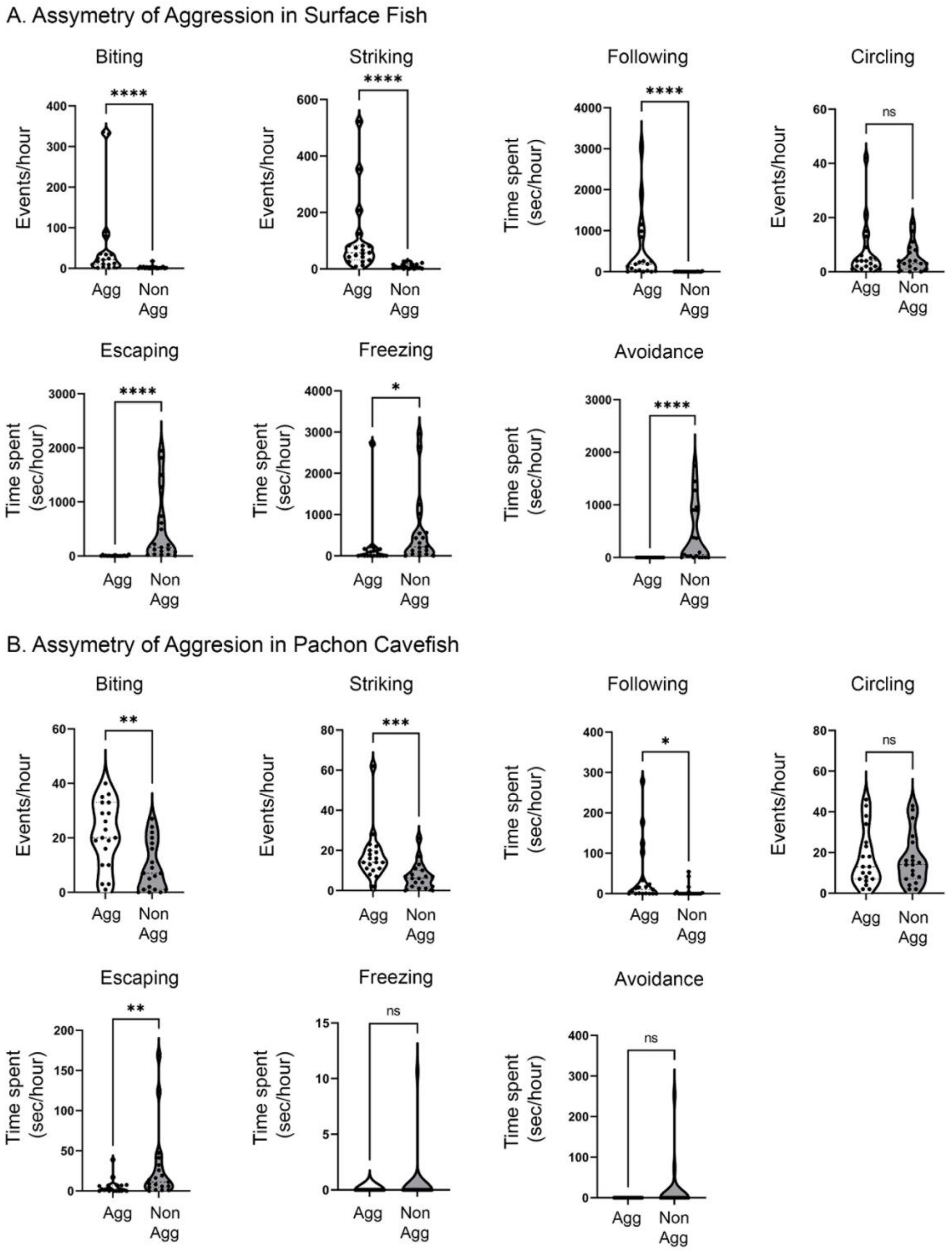

Supplemental Figure 3. Asymmetry of social behaviors between fish during the resident/intruder assay. Quantifications of the differences between designated aggressor/non-aggressor surface fish (A) and aggressor/non-aggressor Pachón cavefish (B) assayed in the resident/intruder assay and pooled in Figure 1. The fish that performed the most striking from each pair, regardless of resident/intruder status, was

designated the “aggressor” fish (Agg), while the other fish in each trial was designated as “non-aggressor” fish (Non Agg). Statistics were performed using non-parametric Mann-Whitneys statistical test for all behaviors. Surface fish: biting ($p<0.0001$), striking ($p<0.0001$), following ($p<0.0001$), circling ($p=0.4991$), escaping ($p<0.0001$), freezing ($p<0.05$), avoidance ($p<0.0001$); Pachón cavefish: biting ($p<0.01$), striking ($p<0.001$), following ($p<0.05$), circling ($p=0.9712$), escaping ($p<0.01$), freezing ($p=0.4872$), avoidance ($p=0.2308$). Significance: $p < 0.05$ (*), $p < 0.01$ (**), $p < 0.001$ (***), $p < 0.0001$ (****), not significant (ns). Statistics can be found in Supplemental Datasheet 3.

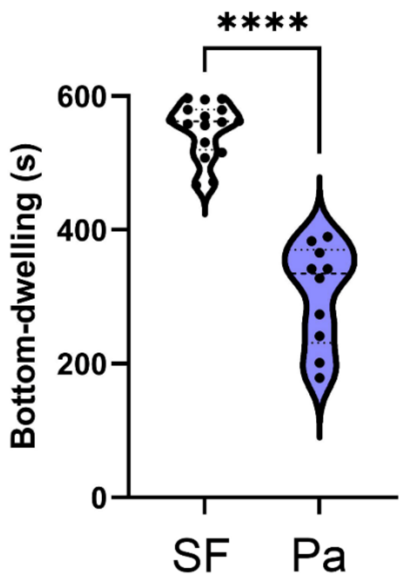

Supplemental Figure 4. Quantification of bottom-dwelling as a measure of stress-like behavior in surface fish (SF) and Pachón (Pa) cavefish using Mann-Whitney U comparison test ($p < 0.0001$). Bottow-dwelling was defined as the time spent in the bottom third of that tank in a 10 min novel tank assay. Significance: $p < 0.0001$ (****).

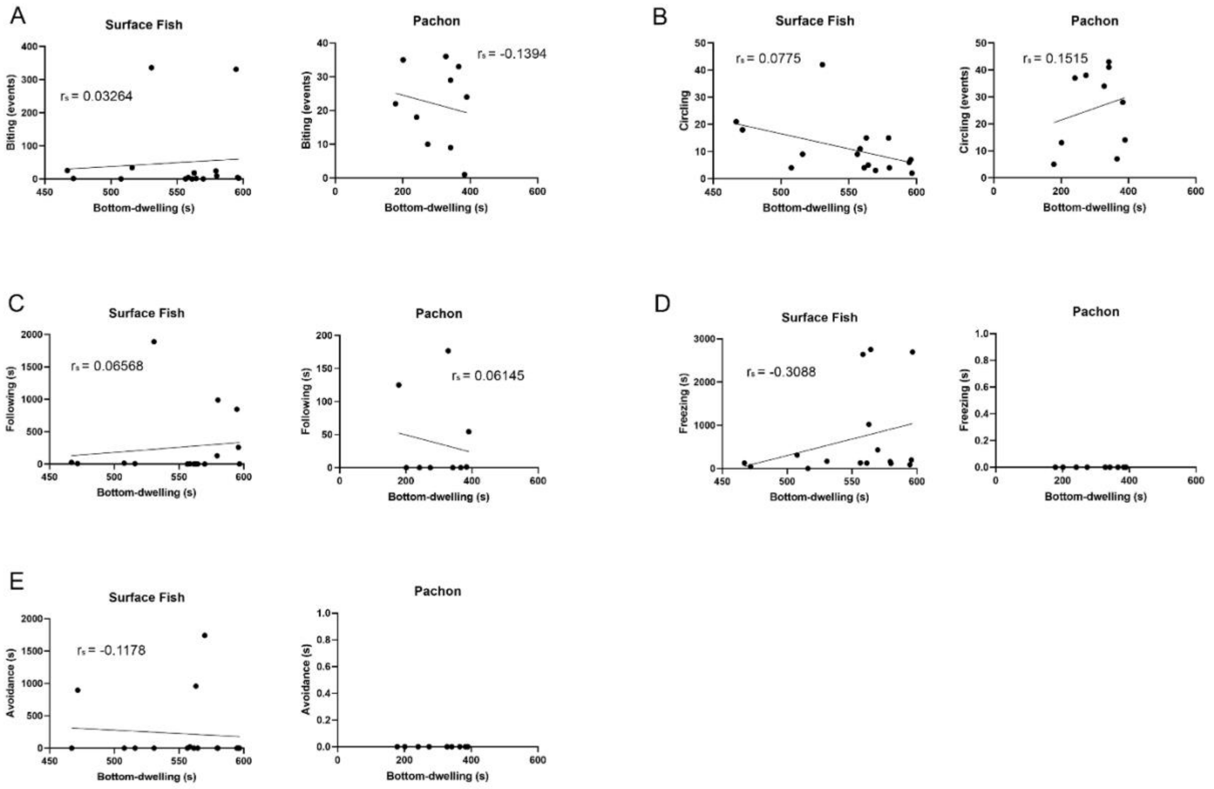

Supplemental Figure 5. Correlation Between Aggression-Associated Behaviors and bottom-dwelling. Correlations in surface fish (left panels) and Pachón (right panels) of time spent bottom-dwelling, defined as time spent in the bottom third of the tank in a novel tank assay, and biting (A, surface, $p = 0.9052$, Pachón, $p = 0.7072$), circling (B, surface, $p = 0.6700$, Pachón, $p = 0.6821$), following (C, surface, $p = 0.8105$, Pachón, $p = 0.8690$), freezing (D, surface, $p = 0.2440$, and avoidance (E, surface, $p = 0.6654$).
